# Botulinum toxin A for primary axillary hyperhidrosis: a narrative review

**DOI:** 10.25122/jml-2026-0005

**Published:** 2026-06

**Authors:** Patcharee Pongsarikan, Pongsiri Koonngam

**Affiliations:** 1 Department of Aesthetic Medicine, College of Integrative Medicine, Dhurakij Pundit University, Bangkok, Thailand

**Keywords:** primary axillary hyperhidrosis, botulinum toxin A, intradermal injection, Hyperhidrosis Disease Severity Scale (HDSS), gravimetric sweat measurement, Dermatology Life Quality Index (DLQI)

## Abstract

Primary axillary hyperhidrosis (PAH) markedly impairs psychosocial well-being and daily functioning. Botulinum toxin type A (BoNT-A) is widely used, both on- and off-label, to suppress eccrine sweating through presynaptic inhibition of acetylcholine release and temporary chemodenervation of cholinergic sympathetic fibers. This narrative review synthesizes published clinical evidence between 2000 and January 2025 regarding the efficacy, durability, safety, and quality-of-life outcomes of BoNT-A for PAH, with comparative consideration of toxin formulations, injection techniques, and device-based alternatives. Evidence from randomized trials, prospective cohorts, long-term observational studies, and meta-analyses consistently demonstrates that intradermal BoNT-A achieves substantial axillary sweat reduction, often exceeding 50–80%, with improvement of at least two grades on the Hyperhidrosis Disease Severity Scale and meaningful reductions in Dermatology Life Quality Index scores. Treatment effects typically persist for approximately 4–7 months per cycle and are associated with predominantly mild, localized adverse events. Comparative data indicate similar efficacy among onabotulinumtoxinA, incobotulinumtoxinA, and abobotulinumtoxinA when equivalent dosing and standardized mapping techniques are applied. Injection strategy, particularly multi-point intradermal delivery guided by starch–iodine mapping, influences coverage and durability. Energy-based modalities—including microwave thermolysis and fractional microneedle radiofrequency—reduce sweating via device-mediated thermal targeting of eccrine glands and show variable comparative outcomes versus intradermal BoNT-A in durability, downtime, and cost. Overall, BoNT-A remains a well-supported first-line injectable therapy for PAH refractory to topical treatment, while further head-to-head and cost-effectiveness studies remain warranted.

## Introduction

Primary axillary hyperhidrosis (PAH) is characterized by excessive sweating beyond thermoregulatory requirements, resulting from hyperactivity of sympathetic cholinergic pathways innervating eccrine sweat glands [1-4]. The condition affects approximately 1–5% of the global population and often presents during adolescence or early adulthood [5-7]. Although not life-threatening, PAH significantly impairs social interactions, occupational performance, and emotional well-being, contributing to anxiety, embarrassment, and reduced quality of life [8].

Conventional first-line treatments such as aluminum chloride antiperspirants and topical glycopyrronium can be effective in mild cases but are frequently limited by local irritation, compliance issues, or suboptimal efficacy [9-11]. Oral anticholinergics, while reducing sweating systemically, may cause undesirable side effects including dry mouth, urinary retention, and blurred vision [12-14]. Consequently, there is a growing demand for minimally invasive, localized therapies that offer sustained efficacy with a favorable safety profile.

Botulinum toxin type A (BoNT-A) acts by inhibiting acetylcholine release at presynaptic terminals of cholinergic neurons, thereby inducing temporary chemodenervation of eccrine glands and effectively reducing sweat secretion in the treated area [15,16]. Since its first approval for cosmetic and neurologic indications, BoNT-A has become a cornerstone in the non-surgical management of focal hyperhidrosis [17-21]. Its use for axillary sites demonstrates reproducible efficacy, high patient satisfaction, and predictable reversibility.

Despite its widespread adoption, several practical aspects remain under continuous investigation. These include the comparative performance of various BoNT-A formulations (onabotulinumtoxinA, incobotulinumtoxinA, abobotulinumtoxinA), optimal dosing per axilla, mapping strategies to ensure uniform coverage, injection depth and spacing, onset and duration of effect, and retreatment intervals necessary to maintain control [22-29]. Furthermore, energy-based modalities, such as microwave thermolysis, radiofrequency microneedling, and focused ultrasound, have emerged as potential alternatives or adjuncts [30-36], with differing mechanisms of action and durability profiles that warrant systematic comparison with intradermal BoNT-A.

Therefore, this narrative review synthesizes published clinical evidence from 2000 to January 2025 regarding BoNT-A therapy for primary axillary hyperhidrosis. It aims to summarize current knowledge on efficacy, durability, safety, and patient-reported outcomes, provide a comparative and mechanism-based perspective across injectable and device-based modalities, identify variations in practice techniques, and highlight knowledge gaps that can inform future research and optimize individualized patient care [37,38].

## Material and Methods

This article was designed as a narrative review aiming to summarize and contextualize available clinical evidence on the efficacy and safety of BoNT-A in the treatment of PAH. A comprehensive literature search was conducted using three major databases—PubMed, ScienceDirect, and Google Scholar—covering publications from January 2000 to January 2025. Search terms combined both Medical Subject Headings (MeSH) and free-text keywords, including “botulinum toxin A,” “axillary hyperhidrosis,” “sweating,” “HDSS,” “gravimetry,” and “quality of life.” Reference lists of included articles were manually screened to identify any additional eligible studies not captured in the initial query.

Inclusion criteria comprised human clinical studies evaluating BoNT-A for PAH and reporting quantitative outcomes such as changes in the Hyperhidrosis Disease Severity Scale (HDSS), gravimetric sweat reduction, or Dermatology Life Quality Index (DLQI) scores. Eligible designs included randomized controlled trials (including split-side comparisons), prospective and retrospective cohort studies, and observational studies. Systematic reviews and meta-analyses that provided relevant quantitative synthesis were incorporated to support contextual interpretation.

Studies were excluded if they focused on non-axillary regions (e.g., palmar, plantar, or craniofacial hyperhidrosis), secondary hyperhidrosis due to systemic disease, investigations involving botulinum toxin type B only, case reports or case series without measurable quantitative outcomes, narrative editorials, or conference abstracts without full data. Only articles published in English were considered to ensure interpretability of outcome measures.

Data from each included study were extracted and organized into summary tables detailing author, year, study design, sample size, intervention dose, injection technique, outcome measures, duration of efficacy, and adverse events. The synthesis was narrative rather than statistical, emphasizing thematic grouping across several domains, formulation type, dosing strategies, injection techniques, durability and retreatment intervals, quality-of-life outcomes, and safety. Because of heterogeneity in study design, outcome definitions, and measurement instruments, no formal meta-analysis was undertaken. The review adhered to academic standards for transparency and reproducibility, although registration in PROSPERO was not applicable due to its narrative nature.

Among all eligible studies, those demonstrating higher methodological rigor (randomized or prospective design), comprehensive quantitative outcome reporting (HDSS, gravimetry, Dermatology Life Quality Index [DLQI]), and clinical representativeness (different BoNT-A formulations and comparators) were prioritized for tabulation. Smaller or technique-focused studies were narratively synthesized but not tabulated to maintain clarity.

## Results

### Study characteristics and evidence base

**Figure 1:**
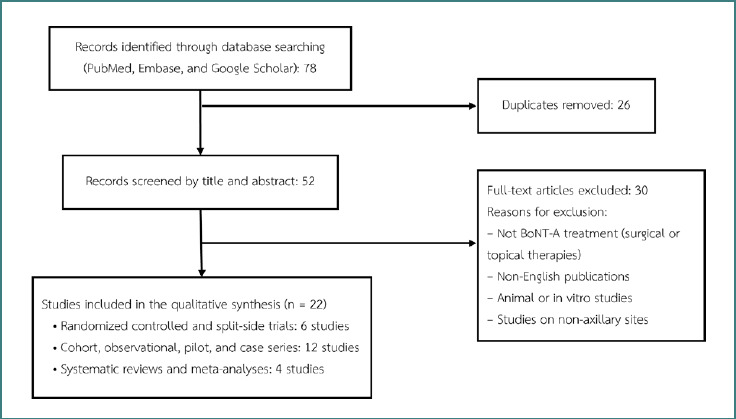
Flow diagram of the study selection process

Between January 2000 and January 2025, the search yielded 78 records; after removing 26 duplicates and screening titles/abstracts, 22 full-text articles met inclusion criteria for narrative synthesis (Figure 1). The corpus spans randomized controlled trials (including split-side designs)[22,34,36], prospective/retrospective cohorts [25,26,39,40], observational series, pilot studies [41,42], and evidence syntheses [21,28]. Sample sizes ranged from small pilot cohorts to phase 3 multicenter randomized controlled trials (e.g., Neu-BoNT/A, *n* = 220)[22] and large long-term clinic cohorts (*n* = 200) [25,26]. Most studies enrolled adults with moderate-to-severe PAH, with selected adolescent data, and followed participants for 3–12 months, while several cohorts extended to 5–10 years. Interventions predominantly used intradermal BoNT-A at 50 U per axilla delivered in 10–25 mapped injections (Minor starch–iodine guidance; 1–2 cm spacing) [24,41]. Alternative formulations (incobotulinumtoxinA, abobotulinumtoxinA, letibotulinumtoxinA, Neu-BoNT/A) and delivery methods (needle-free pneumatic injection, microneedle patch, laser-assisted drug delivery) [35,42,43] were variably represented. Outcomes were consistently assessed using gravimetry, HDSS, DLQI, patient global measures, and adverse-event reporting [21,30,37,39].

A total of 22 studies met inclusion criteria. Table 1 summarizes representative and high-quality studies—primarily randomized and prospective designs with complete quantitative outcome reporting—while smaller or technique-focused reports are discussed narratively.

**Table 1:** Representative clinical studies evaluating botulinum toxin type A (BoNT-A) for primary axillary hyperhidrosis (selected key trials and systematic reviews from 2000–2025; total included = 22 studies)

Study (Year)	Design	*n*	Intervention / Comparator	Follow-up	Main outcomes	Key findings / Comments
**Lee 2022 [22]**	Phase III RCT	220	Neu-BoNT/A 50 U vs Placebo	16 wk	HDSS ↓ ≥ 2 pts 55% vs 6%; DLQI ↑	Effective & safe; median duration ~6 mo
**Castiglione 2024 [39]**	Prospective cohort	81	Inco-BoNT/A 50 U × 2	12 mo	HDSS ↓; DLQI ↓ from 19.9 to 6.9	Sustained 12-mo improvement; no SAE
**Rummaneethorn 2020 [34]**	RCT, split-side	20	BoNT-A 50 U vs FMR	12 wk	HDSS ↓ (p=0.03); DLQI ↓ (p=0.01)	BoNT-A > FMR at all time points
**Grove 2024 [36]**	RCT, split-side	30	BoNT-A vs Microwave Thermolysis	6–12 mo	Gravimetry, odor, QoL	BoNT-A > MWT at 6 mo; similar at 12 mo
**Asfour 2022 [40]**	Prospective	40	Ona-BoNT/A 50 U	16 wk	HDSS ↓ 3 pts; DLQI ↓ 13.5→1	All patients would repeat BTX
**Siri-Archawawat 2023 [41]**	RCT (split-dose)	12	25 U vs 50 U	12 wk	HDSS ↓; QoL ↑	No diff between doses; both safe
**Rosen 2018 [26]**	Long-term cohort	200	Ona-BoNT/A 50 U/axilla (repeated)	10 y	DLQI ↓ 12.2→1.6; HDSS ↓	Durable effect; 97% QoL improvement
**Obed 2021 [21]**	Meta-analysis	937	BoNT-A vs Placebo	2–8 wk	≥ 50% sweat ↓ (RD 0.63); DLQI ↓ 5.5	Significant superiority vs placebo

PAH, primary axillary hyperhidrosis; HDSS, Hyperhidrosis Disease Severity Scale; DLQI, Dermatology Life Quality Index; QoL, quality of life; FMR, fractional microneedle radiofrequency; MWT, microwave thermolysis; U, units; SAE, serious adverse event.

### Efficacy outcomes

Across RCTs and prospective cohorts, BoNT-A produced clinically meaningful sweat reduction and symptom improvement. Placebo-controlled data (phase 3 Neu-BoNT/A) [22,38] demonstrated significant improvement in HDSS responder rates, gravimetric reduction, and global assessments at weeks 4–16. Observational series and prospective cohorts consistently showed ≥50–80% sweat reduction and HDSS improvements of ≥2 points within 2–4 weeks of treatment [25-27,38,40,44]. Dose-ranging and product-comparative studies indicate that 25–50 U per axilla yields robust effects, with several analyses showing no material advantage of 75–100 U over 50 U for most patients [23,41]. Technique studies favored multiple intradermal punctures over radial subcutaneous injection for early and mid-term efficacy, although both techniques demonstrated clinical effectiveness [24]. Overall, efficacy signals were consistent across onabotulinumtoxinA, incobotulinumtoxinA, abobotulinumtoxinA, Neu-BoNT/A, and letibotulinumtoxinA, with no clinically meaningful differences in short-term outcomes when equivalent dosing and standardized mapping strategies were applied [22,23,28,29,41].

### Durability and retreatment

Median duration of effect after a single axillary treatment generally ranged 4–7 months, with several cohorts reporting central tendencies around 6–7 months [25-27,38,44]. Durability often improved modestly with repeated cycles: in long-term series, median inter-treatment intervals lengthened from ~4 months to ~5 months across successive sessions [27], and many patients reported equal or longer benefit with subsequent injections [26]. Predictive analyses suggested that an initial effect lasting ≥6 months portends better long-term persistence and fewer total injections over multi-year horizons [45]. Device-assisted or topical-assisted delivery generally showed shorter persistence than direct intradermal injection, while novel microneedle patches provided encouraging mid-term signals in mild PAH but returned toward baseline by ~24 weeks [35,42].

### Quality of life (QoL) and patient-reported outcomes

BoNT-A translated into substantial improvements in QoL across studies using DLQI, HidroQoL, and patient satisfaction indices. Prospective cohorts reported large DLQI reductions (e.g., median 13.5 → 1 at week 16) and marked shifts from severe to mild symptom categories on HDSS [40]. Multi-year follow-up confirmed durable QoL gains (DLQI ~12 → ~1.6 over >5 years), with high global satisfaction and a sizable proportion describing life-changing benefit [25,26]. Adolescent data mirrored adult findings, with >75% sweat reduction, two-grade HDSS responses in a majority, and sustained improvements through 52 weeks without neutralizing antibodies [44]. Collectively, QoL outcomes were concordant with objective sweat reductions and supported strong patient acceptability across formulations and techniques [22,25-27,37-40, 44].

### Safety and tolerability

Safety profiles were favorable. Adverse events were predominantly mild, transient, and localized—pain, erythema, bruising, pruritus—resolving within days to weeks [24-27,38,39,41, 43,44]. Serious adverse events were not reported in axillary applications across the included studies. Compensatory sweating was uncommon and generally mild when observed in long-term clinical cohorts (≈6–7%) [26]. Comparative product studies (including head-to-head with BoNT-B) and storage-condition analyses (fresh vs frozen reconstituted toxin) showed similar tolerability [46,47]. Long-term series up to 10 years did not identify clinical evidence of tachyphylaxis or systemic toxicity, and repeated treatments remained effective and well-tolerated [25-27,44]. Non-needle delivery (pneumatic, microneedle patch) and laser-assisted methods introduced expected local reactions (erythema, dryness, short-lived discomfort) without sustained sequelae [35,42,43].

### Comparative modalities

Head-to-head randomized split-side trials against fractional microneedle radiofrequency (FMR) showed BoNT-A superiority at 3–12 months in HDSS, objective sweat reduction, QoL, and patient satisfaction [33,34,39]. FMR reduces sweating through controlled dermal thermal injury affecting eccrine glands and adjacent sympathetic fibers, but outcomes appear more technique-dependent than the predictable cholinergic blockade achieved by intradermal BoNT-A.

In comparisons with microwave thermolysis (MWT), BoNT-A achieved superior sweat reduction at approximately 6 months, whereas overall control was comparable by 12 months [30,36]. MWT produces selective thermolytic destruction of eccrine glands at the dermal–subcutaneous junction, offering structural gland modification but with greater procedural invasiveness compared with the reversible neuromodulatory action of BoNT-A.

Laser-assisted drug delivery (fractional CO_2_ with topical BoNT-A) enhanced transdermal penetration but remained inferior to direct intradermal injection at 3–6 months in HDSS, transepidermal water loss, and DLQI [35]. Fractional CO_2_ laser creates microthermal channels that facilitate toxin diffusion, although depth control is less precise than with intradermal injection, which may explain reduced durability. Similarly, dissolvable microneedle platforms provided reduced injection discomfort but shorter durability than conventional intradermal administration [42]. Microneedle systems deliver toxin intradermally through biodegradable projections but may achieve lower effective glandular exposure compared with multi-point injection.

Overall, while energy-based modalities act through structural thermal gland injury, BoNT-A exerts reversible inhibition of acetylcholine release at cholinergic nerve terminals, accounting for differences in durability, invasiveness, and retreatment flexibility [30,34,35,39,42].

## Discussion

This review consolidates two decades of evidence establishing BoNT-A as a cornerstone of non-surgical management for PAH. Across randomized controlled trials, observational studies, and long-term cohorts, BoNT-A consistently demonstrated rapid onset of effect within 2–4 weeks, significant reductions in sweat production exceeding 70–80%, and durable improvement in quality of life lasting approximately 4–7 months [24-27,37-41,43,44]. The therapeutic response was robust across formulations—including onabotulinumtoxinA, incobotulinumtoxinA, abobotulinumtoxinA, and newer preparations such as letibotulinumtoxinA and Neu-BoNT/A—when equivalent dosing and injection mapping were applied [24-27,38,41,43,44]. The most evidence-based protocol involved administration of 50 units per axilla, injected intradermally at 10–15 mapped sites spaced 1–2 cm apart using starch–iodine guidance [24,40,41]. While higher doses occasionally extended duration, they did not substantially improve patient-reported outcomes or overall satisfaction [24,38,41]. Adverse events were minimal, self-limited, and localized, primarily consisting of mild pain, erythema, or bruising at the injection sites [24-27,38-40,44]. Long-term follow-up studies up to 10 years found no tachyphylaxis or systemic toxicity, underscoring the favorable safety profile of BoNT-A for repeated use [25-27,44].

Comparative trials further contextualize the role of BoNT-A among device-based modalities. When compared with FMR, BoNT-A achieved superior sweat reduction and higher patient satisfaction through 3–12 months, although both treatments provided short-term relief [33,34,39]. MWT demonstrated similar efficacy by 12 months, with added benefits such as odor and hair reduction, reflecting its structural ablation of eccrine glands [48]. However, BoNT-A remained preferable for patients prioritizing minimally invasive, reversible treatment with low downtime. Emerging techniques such as laser-assisted BoNT-A delivery, pneumatic jet injection, and microneedle patches have shown promising early outcomes but generally shorter durability than direct intradermal injection [35,36,42]. These innovations may offer alternatives for needle-averse individuals or as adjunctive methods pending further comparative trials. Overall, BoNT-A remains the benchmark for rapid, predictable, and repeatable control of axillary sweating, while energy-based technologies continue to evolve as complementary options for long-term gland modification [33-36,39,42].

Despite strong evidence supporting the efficacy of BoNT-A, several limitations within the literature warrant attention. Many studies employed small sample sizes, unblinded designs, or short follow-up periods, limiting long-term comparative insight [24,33,34,39]. Heterogeneity in injection technique, dilution, and mapping strategy also complicates direct comparisons. Cost-effectiveness data and quality-adjusted life-year analyses remain scarce, despite the widespread clinical use of BoNT-A. Additionally, data in adolescents, high-severity phenotypes, and multimorbid populations are limited, as are investigations into immunogenicity and the potential impact of repeated long-term use across formulations [22,25-27,44]. Future research should prioritize adequately powered head-to-head randomized controlled trials comparing BoNT-A with MWT and FMR over at least 12–24 months using standardized gravimetric and patient-reported endpoints [33,34,36,39]. Dose–technique optimization studies stratified by sweat map phenotype could refine injection grids and reduce total toxin use without compromising durability [24,40,41]. Economic and cost–utility analyses are also critical to inform policy and payer decisions, particularly in resource-limited settings. Furthermore, innovations in delivery systems—such as microneedle arrays and needle-free pneumatic devices—should be evaluated in non-inferiority frameworks to determine whether they can provide equivalent outcomes with reduced discomfort and downtime [35,42]. Expanding data on special populations, including adolescents and individuals with underlying systemic conditions, will further ensure equitable access and safety across diverse patient groups [22,25,26,44].

In conclusion, intradermal BoNT-A—administered at approximately 50 units per axilla with systematic, starch–iodine-guided, multi-point injection—offers reproducible, safe, and patient-centered symptom control for primary axillary hyperhidrosis [24-27,37-41,43,44]. Its consistent short- to mid-term efficacy, strong safety record, and high satisfaction rates solidify its role as the first-line injectable therapy. Energy-based procedures, particularly microwave thermolysis (MWT), provide valuable alternatives for patients seeking longer-term structural outcomes, while novel transdermal and microneedle technologies represent an emerging frontier [30,33-35,39,42]. Addressing the remaining evidence gaps through rigorous, long-term, and comparative trials will refine treatment algorithms, optimize durability per unit dose, and strengthen the integration of BoNT-A into comprehensive, cost-effective hyperhidrosis care.

## Personal thanks

The authors would like to acknowledge academic guidance and institutional support from the College of Integrative Medicine, Dhurakij Pundit University, which facilitated the development of this review.
